# Towards the development of a core outcome set for post-stroke facial palsy (COS post-stroke facial palsy): a study protocol for establishing professional consensus on what to measure

**DOI:** 10.1186/s13063-026-09556-z

**Published:** 2026-03-05

**Authors:** Havva Sumeyye Eroglu, Audrey Bowen, Matthew Checketts, Claire Mitchell

**Affiliations:** 1 Division of Psychology, Communication and Human Neuroscience, Faculty of Biology, Medicine and Health, University of Manchester, Room G700, Stopford Building, Oxford Road, Manchester, M13 9PL England, UK; 2 Division of Psychology and Mental Health, Faculty of Biology, Medicine and Health, The University of Manchester, Manchester, UK; 3 School of Psychology & Neuroscience, University of Glasgow, Glasgow, UK; 4 Geoffrey Jefferson Brain Research Centre, The Manchester Academic Health Science Centre, Northern Care Alliance & University of Manchester, Manchester, UK

**Keywords:** Stroke, Facial palsy, Core outcome set, Delphi process

## Abstract

**Background:**

Facial palsy affects 45–60% of acute stroke patients, significantly impacting their physical function, communication, and quality of life. Varied outcome measures across studies and clinical practices make it difficult to synthesise evidence and establish treatment effectiveness. This protocol describes in detail the professional stakeholder component of a multistage Core Outcome Set (COS) development programme for post-stroke facial palsy. This component focuses on identifying critically important outcomes (the “what” to measure) between two key stakeholder groups: clinicians and researchers.

**Methods:**

This study follows the Core Outcome Set standards for development (COS-STAD) and protocol (COS-STAP) recommendations. The development process consists of five sequential steps: (1) outcome generation through systematic reviews, consultation with our co-researchers who have lived experience, and the findings from qualitative interviews with stroke participants; (2) recruitment of up to 200 international clinicians and researchers (minimum 30 per group) through clinical networks (including professional associations), social media platforms (X, LinkedIn, Bluesky), and via published research identifying stroke and facial palsy researchers; (3) multiple rounds of Delphi surveys; (4) online consensus meeting (at least five per stakeholder group); and (5) results dissemination.

**Discussion:**

This paper reports the detailed protocol for the professional stakeholder component, which will establish consensus among clinicians and researchers on what outcomes are critically important to measure. This protocol addresses a significant gap in stroke rehabilitation research as part of a comprehensive, multi-stage programme to develop a COS for post-stroke facial palsy. Standardising outcome measurement will facilitate more effective synthesis of research findings, reduce research waste, and accelerate intervention development. It describes how people with lived experience have been included from the start and signposts to a parallel study using the supported nominal group technique (detailed protocol reported separately). A parallel systematic review will evaluate available measurement instruments, their psychometric properties to explore “how” to measure the agreed outcome domains. The results of each stage will be disseminated through multiple channels to facilitate widespread research and clinical practice adoption.

**Trial registration:**

Core Outcome Measures in Effectiveness Trials (COMET) registered, December 2024 (https://www.comet-initiative.org/Studies/Details/3295); systematic review registered (PROSPERO), June 2023, CRD42023410768.

**Supplementary Information:**

The online version contains supplementary material available at 10.1186/s13063-026-09556-z.

## Background

Facial palsy is characterised by partial or complete loss of voluntary and involuntary facial muscle control caused by upper or lower motor neuron lesions [1, 2]. Facial palsy due to an upper motor neuron lesion is a common consequence of stroke [3–5], affecting 45–60% of acute stroke patients [6–8]. Facial palsy affects physical appearance, communication, speech, eating, and swallowing [9, 10], having a detrimental impact on stroke survivors’ well-being and quality of life [11].

Systematic reviews on post-stroke facial palsy revealed the use of widely varying outcome measures [5, 12]. While only the Sunnybrook facial grading system has psychometric data for post-stroke facial palsy [13], a variety of measures are used in clinical practice. A recent survey of allied health professionals revealed that informal oromotor examination (40%) and the Sunnybrook Facial Grading Scale (22%) were the most frequently used assessment tools in clinical practice, with additional varied measures including the Facial Disability Index, Facial Clinimetric Evaluation Scale, and EQ-5D-5L [3]. This heterogeneity in outcome measurement makes it difficult to synthesise evidence and establish treatment effectiveness, thereby hindering meaningful comparison of interventions and contributing to research waste [12, 14].

A Core Outcome Set (COS) is defined as a standardised set of outcomes that should be measured and reported, as a minimum, in all studies for a specific health condition [15]. The importance of developing such standardised outcome sets has been highlighted across various clinical fields [16–18]. The development of a COS for post-stroke facial palsy would enable the combination and comparison of findings across studies, reduce reporting bias, and ensure that collected data are both useful and usable. This standardisation can help clinicians and researchers to understand what outcomes are important to measure in clinical practice and research.

The development of a COS for post-stroke facial palsy is a multi-stage, overarching programme of work involving multiple stakeholder groups and methodological approaches. The aim of this paper is to report the protocol for the development of an international consensus-based COS for post-stroke facial palsy, focusing on identifying what should be measured in research from the perspective of two of the three key stakeholder groups, researchers, and healthcare professionals. These two groups have specialist knowledge of outcome use, interpretation, and feasibility in research and clinical contexts. They are well placed to identify relevant outcome domains based on their experience of selecting, using, and evaluating outcomes across existing research and clinical practice. The third stakeholder group, people with lived experience, should be included from the outset. As part of this, a parallel study with stroke survivors using the supported nominal group technique will be reported separately to ensure a detailed description of accessible, rigorous participation methods. The voice of the lived experience group plays an important role in identifying key outcomes for research, and this perspective often differs from or complements that of academic researchers and other stakeholders [19]. Evidence demonstrates that outcomes identified as important by people with lived experience are often not found in the clinical literature [20]. People with lived experience have shown preferences for self-reported outcomes that capture their lived reality, which may differ from outcomes prioritised by clinicians [19, 20]. This underscores why lived experience input must be captured as core data throughout the COS development process. The subsequent step of determining how these outcomes should be measured will be addressed through our systematic review.

### Study objectives

#### Overall COS development programme objective

To develop an international consensus-based COS for post-stroke facial palsy research through a multi-stage programme involving professional stakeholders and people with lived experience.

#### Objectives for this professional stakeholder component

The primary objective of this paper is to describe methods for reaching an international consensus among clinicians and researchers on what outcome domains should be measured in post-stroke facial palsy research.

Specific objectives include the following:

- To identify a comprehensive long list of possible outcome domains.
- To achieve international consensus on what outcomes are considered critically important to measure among clinicians and researchers.

This protocol focuses on the long list generation and Delphi study with professional stakeholders. A separate protocol will address the parallel study with people with lived experience using nominal group technique, and a protocol of our systematic review of measurement instruments has been registered on PROSPERO (CRD42023410768).

Principal Research Question:

What are the most important outcomes to measure for people with post-stroke facial palsy, as determined by international consensus among clinicians and researchers?

## Methods

This protocol was developed in accordance with two key methodological frameworks: the Core Outcome Set standards for development (COS-STAD) recommendations, the Core Outcome Set standardised protocol (COS-STAP) checklist and Standard Protocol Items: Recommendations for Interventional Trials (SPIRIT) reporting guidelines [21–23]. The completed COS-STAP and SPIRIT checklists are provided as supplementary documents (Additional file 1 and Additional file 2, respectively).

### Scope

This initial phase of COS development addresses facial palsy occurring after stroke in adults (aged ≥ 18 years), regardless of stroke type or time since onset. The COS will be applicable across all intervention types. While primarily intended for research applications (clinical trials, observational studies, and systematic reviews), the translation of COS to clinical practice presents distinct implementation challenges, with evidence suggesting variable uptake and impact across different healthcare settings [24].

### Ethics and consent

Research ethics approval was obtained from the University of Manchester (reference 2024–21316-38,685).

Informed consent will be obtained for participation using different approaches appropriate to each study component. For the e-Delphi surveys, informed consent will be obtained through an explicit consent process on the first page of the online survey: participants will be directed to read the participant information sheet via a provided link and must actively select “yes” to confirm they have read and understood the information and consent to proceed with the survey. This digital consent process ensures informed participation while being appropriate for the online survey format. Written consent will be obtained from the subset participating in the consensus meeting.

### Study design

**Fig. 1:**
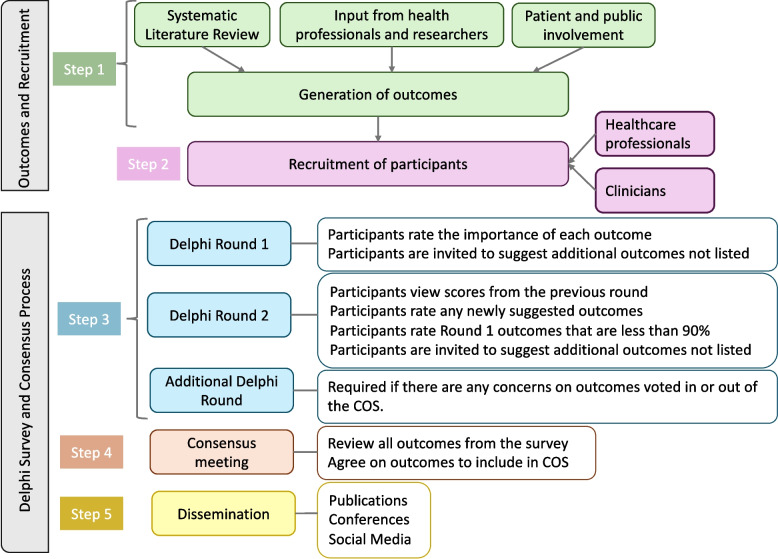
Workflow for the initial phase of developing the COS study process

This initial phase to develop a COS for post-stroke facial palsy has been developed using a multiple-method design incorporating a modified Delphi process with online surveys and consensus meetings, as illustrated in Fig. 1. The study will be conducted online, allowing for international participation without geographical restrictions from healthcare professionals and researchers recruited through various clinical networks, research collaborations, and professional associations worldwide. The modified Delphi process methodology has been chosen to establish consensus among two key groups of stakeholders, clinicians and researchers. The development process consists of five sequential steps, which are described in detail below: outcome generation and recruitment, followed by Delphi survey rounds, consensus meeting, and dissemination of results.

This approach follows the Cochrane Skin–Core Outcome Set Initiative (CS-COUSIN) roadmap, a framework for developing core sets of outcomes and measurements [25]. While this framework was originally developed for dermatology research, its methodological principles are applicable across clinical conditions, and we have adapted its systematic approach by tailoring the scope, stakeholder groups, and outcome processes to suit the specific context of post-stroke facial palsy research.

### Step-1: generation of outcomes

The initial phase of developing our COS for post-stroke facial palsy involves creating a comprehensive long list of potential outcome domains through multiple sources. We will include outcomes that were used in efficacy and effectiveness trials included in existing systematic reviews of post-stroke facial palsy [5, 12], and we will update these searches to ensure that more recent trials are also captured.

To ensure the inclusion of patient-relevant outcomes, we will work closely with a stroke survivor lived experience (patient and public involvement, PPI) group. This group comprises stroke survivors with experience of post-stroke facial palsy who have been involved in our wider programme of work and have experience of developing other core outcome sets. Our approach to PPI follows established principles for meaningful patient involvement in research [26], ensuring stroke survivors contribute substantively to outcome identification and framing rather than simply reviewing researcher-generated lists. This is essential for capturing outcomes reflecting life impact [27].

At the earliest stage of this project, before the Delphi surveys are launched, the PPI group will: (a) review the preliminary list of outcomes identified from the systematic reviews, (b) consider the early findings from planned qualitative interviews to explore impact and what is important to people with facial palsy after stroke, (c) identify missing concepts and propose additional outcomes that reflect any other priorities, and (d) advise on the wording and framing of outcomes to ensure they are understandable and meaningful to stroke survivors. This pre-Delphi work ensures that patient-identified outcomes, patient language, and patient perspectives on outcome importance inform the professional Delphi process and contents [28]. Feedback from the PPI group will be discussed by the study team and incorporated into a revised long list of potential outcome domains, which will form the basis of the Delphi survey for clinicians and researchers. In parallel, stroke research colleagues will be consulted to check for any further outcomes domains that may have been overlooked. The consolidated long list will then be systematically categorised according to the International Classification of Functioning, Disability and Health (ICF) framework [29], organising outcome domains into distinct levels: body structures and functions, activity, participation, and wellbeing/quality of life.

### Step-2: recruitment

This study will target two stakeholder groups with the following inclusion criteria: (1) researchers who are or who have been involved in stroke or facial palsy related research, (2) healthcare professionals with experience of working with patients who have post-stroke facial palsy. The recruitment process will extend internationally to maximise the breadth and depth of professional input. While healthcare professionals from all disciplines are eligible, we anticipate higher representation from speech and language therapists and physiotherapists, as these professionals are typically most involved in the direct management of post-stroke facial palsy [30]. The study aims to recruit a diverse group of professionals across various geographical regions and professional backgrounds, with varied expertise in facial palsy research and clinical practice, to ensure comprehensive insights into what is important to measure for people living with post-stroke facial palsy. Participants will complete the survey online, which includes both demographic questions (e.g. professional background, years of experience) and the Delphi outcome rating questions, at a time convenient for them.

There is no universally agreed optimal number of participants for a Delphi study, and sample sizes can vary widely [31]. We aim to recruit up to 200 participants across two stakeholder groups: 100 clinicians and 100 researchers. Our minimum target is 30 participants per group, which aligns with previous COS studies [32]. We expect that at least 13 participants from each group will complete the final round of the Delphi survey, consistent with other COS developments [33]. Participants will be sent the participant information, confirm they have read this, and give consent to continue to the online survey; all personal data will be stored securely and pseudonymised.

We intend to recruit healthcare professionals from various clinical networks of healthcare professionals working in facial palsy and stroke, including professional associations across rehabilitation, neurology, physiotherapy, speech and language therapy, occupational therapy, orthoptics, nursing, medicine, and psychology (e.g. Facial Therapy Specialists International, World Federation for NeuroRehabilitation, World Stroke Organization, Association of Chartered Physiotherapists Interested in Neurology, International Orthoptic Association, Psychological Society of Ireland, Swedish Association of Occupational Therapists, Federazione Logopedisti Italiani, British and Irish Association of Stroke Physicians, National Stroke Nursing Forum). We will also advertise the study via our dedicated study accounts on social media platforms (X and Bluesky) and through posts on study team members’ LinkedIn profiles. For researcher recruitment, we will contact researchers identified through systematic reviews of post-stroke facial palsy and other published studies [5, 12, 34–36]. In addition, we will use our study accounts on X and Bluesky and study team members’ LinkedIn profiles to share the recruitment invitation. While some researchers may also be healthcare professionals, in this study we are categorising “researchers” as those primarily engaged in academic or research activities, as distinct from those participating primarily in a clinical capacity. Demographic data will be collected in the survey allowing participants to indicate whether they identify as a researcher, clinician, or both.

The first round of Delphi survey participant recruitment has commenced. At the time of protocol submission, participant recruitment and data collection are actively ongoing. If recruitment targets are not met, the recruitment period may be extended. The consensus meeting will begin after the completion of the Delphi survey rounds.

### Step-3: delphi survey

Using the outcomes identified during Step 1, participants will complete the first round of the online Delphi survey by rating the importance of each outcome on a 1–9 scale (1 = least important, 9 = most important).

Rankings of 7–9 indicate critical importance, 4–6 outcomes that are important but not critical, whilst ratings of 1–3 are of limited importance using the Grading of Recommendations Assessment, Development, and Evaluations (GRADE) scale [37].

To minimise the impact of missing data, questions within each domain will be presented in random order for each participant, consistent with evidence showing the influence of question order in outcome prioritisation [38].

The consensus criteria for including or excluding outcomes in the COS for post-stroke facial palsy will be predefined. An outcome will be considered to have reached “consensus in” (of the COS) when 70% or more of participants in each stakeholder group rate it as critically important (scoring 7–9) and fewer than 15% rate it as having limited importance (scoring 1–3). Conversely, an outcome will be considered to have reached “consensus out” when 50% or fewer participants in each stakeholder group rate it as critically important (scoring 7–9). All outcomes that are clearly “consensus in” or “consensus out” from the survey will be briefly ratified during the consensus meeting. The outcomes with no clear consensus either way from the survey will be discussed in more detail at the consensus meetings. This definition ensures that the COS includes outcomes that are consistently rated as highly important across all stakeholder groups while excluding those with limited perceived importance.

Participants will be invited to suggest additional outcomes that they consider important but had not been included in the round 1 Delphi survey. These newly suggested outcomes will be reviewed by the study team. The team will discuss each suggestion in detail to determine whether it is already captured by an existing outcome domain or represents a genuinely new outcome. Where suggestions are not adequately covered by existing outcomes, they will be added to round 2 of the Delphi survey for participant rating. After completing round 1, all responses will be analysed to assess differences in priority. All completed outcome ratings will be analysed anonymously according to each stakeholder group. If a participant rated some outcomes but did not complete the survey, their available ratings will be included in the analysis. However, completely blank responses will be excluded. If 90% or above of both stakeholder groups rate any outcomes as either 7–9 critically important or 1–3 limited importance, these will be removed from round 2 of the survey but will still be ratified or discussed in the consensus meeting as appropriate. Participants will be informed of any changes to the survey. In addition, we will consider comments from participants completing round 1, which may involve adding new outcomes or removing others.

The survey will include basic demographic questions (gender, ethnicity, country of residence, and duration of professional experience) along with the main survey questions. These demographic data will be collected to characterise the diversity and representativeness of our participant sample.

In round 2 of the Delphi process, participants will see the percentage distribution of scores from both stakeholder groups and their own previous ratings. In keeping with Delphi methodology principles, participants will only be presented with anonymised, aggregated responses during subsequent survey rounds [39]. Participants will be notified of any modifications made based on round 1 feedback. After reviewing this feedback, participants will be asked to re-rate relevant outcomes, considering both their initial response and the collective responses from all stakeholder groups. Participants will again have the opportunity to suggest additional outcomes, as in round 1, which, if appropriate, will be included for rating if we proceed to round 3.

If after round 2, there are clear discrepancies between the two groups where one group is strongly “consensus in” or “consensus out” and this is the direct opposite of the other group, a third round of the survey might be appropriate. This additional round will follow the same format as round 2, with participants viewing the updated group responses and their previous ratings before re-rating these remaining outcomes. Ethics approval is in place for this potential additional round if required.

All survey rounds will be conducted online via Qualtrics and will remain open for 4–6 weeks each. If participation rates are low, emails will be sent to the various networks to encourage participation. If subsequent survey response rates are low, then participants will be sent reminders at the end of week 2 to complete the survey at the email addresses they registered in round 1 [40]. The responses from healthcare professionals and researchers will be analysed separately to identify any differences in priorities.

At the end of each round, participants will be asked to provide their email addresses to allow us to invite them to the subsequent Delphi survey rounds and the consensus meeting. Contact information will be stored separately and securely from survey responses in accordance with data protection regulations and to ensure anonymised analysis.

### Step-4: consensus meeting

Following the Delphi rounds, an online consensus meeting will be held to review survey results and establish agreement on the COS. To ensure effective group discussion, participation will be limited to 10 participants from each stakeholder group (with at least five participants per group), purposively selected from those who completed all Delphi rounds to maintain a balanced representation of both stakeholder groups (approximately equal numbers of researchers and clinicians) and geographical regions. All participants will be required to provide written consent and verify their professional credentials prior to participation. The consensus meeting will be conducted via Microsoft Teams, a secure video conferencing platform. Participants will be asked to briefly identify themselves at the start of the meeting but will have the option to turn off their video thereafter. The meeting will be recorded on an encrypted device for data analysis purposes, with recordings deleted following analysis. Voting will be conducted anonymously within Microsoft Teams using yes/no polls, and the results of each vote will be securely recorded for analysis.

The meeting will be led by an independent facilitator who will present the results from all Delphi rounds according to the predefined consensus criteria.

For outcomes that were voted by both groups as “consensus out” during the Delphi rounds, the facilitator will briefly ask if anyone has compelling reasons to re-visit any of these items. If not, these outcomes will be ratified, then excluded. If participants express doubts, a structured discussion will be held to allow views to be shared among the group. Following the discussion, the outcome will be taken forward to a yes/no vote. Outcomes receiving less than 70% “yes” votes will be excluded, while those reaching the 70% threshold will be included in the final COS for post-stroke facial palsy.

For outcomes that reached “consensus in” during the survey, they would then be discussed as to whether they are still considered critically important to be included in the COS post-stroke facial palsy. Voting would have to reach the 70% threshold of “yes” votes required for inclusion in the COS.

For outcomes that did not reach either “consensus in” or “consensus out”, there would need to be more detailed discussion in the meeting, and this discussion would help the participants in the consensus meeting decide which way to vote. All participants will then vote on whether each outcome should be included (“yes”) or excluded (“no”) from the COS, with a 70% threshold of “yes” votes required for inclusion in the COS.

### Step-5: dissemination

We will adopt a multi-method approach to dissemination of this first stage to develop a COS-post stroke facial palsy. Reporting will follow the COS-STAR (Core Outcome Set-STandards for Reporting) guidelines [40]. The initial findings for the post-stroke facial palsy COS will be published in an open-access journal, presented at conferences, and shared through professional networks (e.g. special interest groups and clinical excellence networks) to maximise reach among researchers and clinicians. To ensure accessibility for people with lived experience, we will also disseminate results via meetings with stroke survivors, our PPI group, stroke charities, support organisations, and online platforms, using formats such as plain language summaries and infographics. After publication, our initial COS for post-stroke facial palsy will be available on the Core Outcome Measures in Effectiveness Trials (COMET) database and has already been registered with COMET (https://www.comet-initiative.org/Studies/Details/3295).

## Discussion

This protocol outlines our methods for the professional stakeholder component of a multi-stage programme to develop a COS for post-stroke facial palsy. Our approach includes online Delphi surveys for initial agreement followed by a consensus meeting for final decision-making to agree what is critically important to measure in post-stroke facial palsy according to two of the three key stakeholder groups, researchers and clinicians working in this area. Throughout this process, we will strictly adhere to published standards for core outcome development in stroke and facial palsy research and follow established methodological guidance [21, 40].

This starting point of COS development addresses a critical gap in stroke and facial palsy rehabilitation research. A final standardised outcome set will facilitate several important advances in the field. First, it will enable more effective synthesis of future research findings, allowing for meaningful comparisons across studies. Second, it will optimise resource allocation in research by ensuring that essential outcomes are consistently measured; or where relevant measurement instruments do not exist, it will indicate a need for this development. These improvements will ultimately accelerate the development and evaluation of intervention approaches for facial palsy management.

Our systematic and structured approach includes multiple steps. Through this initial step of the work, we will establish preliminary core outcome domains through a comprehensive literature review and expert consultation with healthcare professionals and researchers, front-loaded by input from people with lived experience through qualitative interviews and Patient and Public Involvement [26].

People with lived experience (stroke survivors) are core stakeholders in this COS development programme. Rather than join this Delphi process, their participation in rating and reaching consensus on outcome domains requires different, more inclusive methodology to ensure accessible and supported participation. Many stroke survivors experience communication difficulties, cognitive changes, or fatigue, which necessitate careful methodological planning. A parallel study with stroke survivors using a supported nominal group technique is planned and will be reported in a separate, dedicated protocol paper. Nominal group technique is particularly appropriate for people with communication difficulties as its structured, turn-taking format supports equal participation and allows time for communication facilitation [41]. This approach ensures that lived-experience perspectives are captured using rigorous, appropriate methods with accessible materials and appropriate support for communication and participation.

In parallel, we are conducting a systematic review of outcome measures, which will provide crucial information about what measures are available that fit the agreed domains, their psychometric data, and clinical utility/practical implementation. This will help evaluate and recommend appropriate measurement instruments for each outcome and inform the future development of a core measurement set (completing the “what” to measure and the “how” to measure it).

## Trial status

This protocol (version 8.0, dated February 14, 2025) describes the development of a COS that is ongoing. The first round of Delphi is January–February 2025, the second round is April 2025, the third round (if necessary) is May 2025, and consensus meetings are June 2025.

## Supplementary Information

Supplementary file1. SPIRIT 2025 checklist (DOCX 35.5 KB)

Supplementary file2. COS-STAP statement checklist (DOCX 20.0 KB)

## Data Availability

Anonymised data from this study will be available on reasonable request up to 5 years in accordance with our ethics approval. Requests should be directed to the corresponding author (havvasumeyye.eroglu@postgrad.manchester.ac.uk) or to Claire Mitchell (claire.mitchell@manchester.ac.uk).

## Abbreviations

COS: Core Outcome Set
COMET: Core Outcome Measures in Effectiveness Trials
COS-STAP: Core Outcome Set-Standardised Protocol Items
COS-STAD: Core Outcome Set standards for development recommendations
COS-STAR: Core Outcome Set-Standards for Reporting guidelines
CS-COUSIN: Cochrane Skin – Core Outcome Set Initiative
SPIRIT: Standard Protocol Items: Recommendations for Interventional Trials
PPI: Patient and public involvement
ICF: International Classification of Functioning, Disability and Health

## References

[1] Alberstone CD, Benzel EC, Najm IM, Steinmetz MP. Anatomic basis of neurologic diagnosis. 2nd ed. New York: Thieme; 2009. p. 252–5.

[2] Wilson-Pauwels L, Stewart PA, Akesson EJ, Spacey SD. Cranial nerves: function and dysfunction. 3rd ed. Shelton (CT): PMPH USA; 2010. p. 115–7.

[3] Vaughan A, Copley A, Miles A. Physical rehabilitation of central facial palsy: a survey of current multidisciplinary practice. Int J Speech Lang Pathol. 2022;24(6):616–25. 34928754 10.1080/17549507.2021.2013533

[4] Silva MC, Oliveira MT, Azevedo-Santos IF, DeSantana JM. Effect of proprioceptive neuromuscular facilitation in the treatment of dysfunctions in facial paralysis: a systematic literature review. Braz J Phys Ther. 2022;26(6):100454. 36279766 10.1016/j.bjpt.2022.100454 PMC9597113

[5] Fabricius J, Kothari SF, Kothari M. Assessment and rehabilitation interventions for central facial palsy in patients with acquired brain injury: a systematic review. Brain Inj. 2021;35(5):511–9. 33645363 10.1080/02699052.2021.1890218

[6] Cattaneo L, Pavesi G. The facial motor system. Neurosci Biobehav Rev. 2014;38:135–59. 24239732 10.1016/j.neubiorev.2013.11.002

[7] Mitchell C, Gittins M, Tyson S, Vail A, Conroy P, Paley L, et al. Prevalence of aphasia and dysarthria among inpatient stroke survivors: describing the population, therapy provision and outcomes on discharge. Aphasiology. 2021;35(7):950–60.

[8] Volk GF, Steinerstauch A, Lorenz A, Modersohn L, Mothes O, Denzler J, et al. Facial motor and non-motor disabilities in patients with central facial paresis: a prospective cohort study. J Neurol. 2019;266(1):46–56. 30367260 10.1007/s00415-018-9099-x

[9] Konecny P, Elfmark M, Urbanek K. Facial paresis after stroke and its impact on patients’ facial movement and mental status. J Rehabil Med. 2011;43(1):73–5. 21174055 10.2340/16501977-0645

[10] Movérare T, Lohmander A, Hultcrantz M, Sjögreen L. Peripheral facial palsy: speech, communication and oral motor function. Eur Ann Otorhinolaryngol Head Neck Dis. 2017;134(1):27–31. 27836742 10.1016/j.anorl.2015.12.002

[11] Schimmel M, Ono T, Lam O, Müller F. Oro‐facial impairment in stroke patients. J Oral Rehabil. 2017;44(4):313–26. 28128465 10.1111/joor.12486

[12] Vaughan A, Gardner D, Miles A, Copley A, Wenke R, Coulson S. A systematic review of physical rehabilitation of facial palsy. Front Neurol. 2020;11:222. 32296385 10.3389/fneur.2020.00222 PMC7136559

[13] Tramontano M, Morone G, La Greca FM, Marchegiani V, Palomba A, Iosa M, et al. Sunnybrook facial grading system reliability in subacute stroke patients. Eur J Phys Rehabil Med. 2021;57(5):685–90. 34105919 10.23736/S1973-9087.21.06629-6

[14] Ioannidis JP, Greenland S, Hlatky MA, Khoury MJ, Macleod MR, Moher D, et al. Increasing value and reducing waste in research design, conduct, and analysis. Lancet. 2014;383(9912):166–75. 24411645 10.1016/S0140-6736(13)62227-8 PMC4697939

[15] Clarke M. Standardising outcomes for clinical trials and systematic reviews. Trials. 2007;8(1):39. 18039365 10.1186/1745-6215-8-39 PMC2169261

[16] Webbe JWH, Duffy JM, Afonso E, Al-Muzaffar I, Brunton G, Greenough A, et al. Core outcomes in neonatology: development of a core outcome set for neonatal research. Arch Dis Child Fetal Neonatal Ed. 2020;105(4):425–31. 31732683 10.1136/archdischild-2019-317501 PMC7363790

[17] Sinha IP, Gallagher R, Williamson PR, Smyth RL. Development of a core outcome set for clinical trials in childhood asthma: a survey of clinicians, parents, and young people. Trials. 2012;13(1):103. 22747787 10.1186/1745-6215-13-103 PMC3433381

[18] Fang G, Yu W, Chen D, Ding X, Qiao L, Zhang L, et al. Development of a core outcome set of clinical research on the integration of traditional Chinese and Western medicine for spinal metastases: a study protocol. BMJ Open. 2024;14(9):e083315. 39260838 10.1136/bmjopen-2023-083315 PMC11409365

[19] Raval P, Moreno F, Needleman I. Patient involvement to explore research prioritisation and self-care management in people with periodontitis and diabetes. Br Dent J. 2021. 10.1038/s41415-021-3175-9. 10.1038/s41415-021-3175-9 34239054

[20] Crawford MJ, Robotham D, Thana L, Patterson S, Weaver T, Barber R, et al. Selecting outcome measures in mental health: the views of service users. J Ment Health. 2011;20(4):336–46. 21770782 10.3109/09638237.2011.577114

[21] Kirkham JJ, Davis K, Altman DG, Blazeby JM, Clarke M, Tunis S, et al. Core outcome Set-STAndards for development: the COS-STAD recommendations. PLoS Med. 2017;14(11):e1002447. 29145404 10.1371/journal.pmed.1002447 PMC5689835

[22] Kirkham JJ, Gorst S, Altman DG, Blazeby JM, Clarke M, Tunis S, et al. Core outcome Set-STAndardised protocol items: the COS-STAP statement. Trials. 2019;20(1):116. 30744706 10.1186/s13063-019-3230-x PMC6371434

[23] Chan A-W, Tetzlaff JM, Gøtzsche PC, Altman DG, Mann H, Berlin JA, et al. SPIRIT 2013 explanation and elaboration: guidance for protocols of clinical trials. BMJ. 2013;346:e7586. 23303884 10.1136/bmj.e7586 PMC3541470

[24] Chiarotto A, Ostelo RW, Turk DC, Buchbinder R, Boers M. Core outcome sets for research and clinical practice. Braz J Phys Ther. 2017;21(2):77–84. 28460714 10.1016/j.bjpt.2017.03.001 PMC5537457

[25] Schmitt J, Apfelbacher C, Spuls PI, Thomas KS, Simpson EL, Furue M, et al. The Harmonizing Outcome Measures for Eczema (HOME) roadmap: a methodological framework to develop core sets of outcome measurements in dermatology. J Invest Dermatol. 2015;135(1):24–30. 25186228 10.1038/jid.2014.320

[26] Young B, Bagley H. Including patients in core outcome set development: issues to consider based on three workshops with around 100 international delegates. Res Involv Engagem. 2016;2(1):25. 29507761 10.1186/s40900-016-0039-6 PMC5831887

[27] Dodd S, Gorst SL, Young A, Lucas SW, Williamson PR. Patient participation impacts outcome domain selection in core outcome sets for research: an updated systematic review. J Clin Epidemiol. 2023;158:127–33. 37054902 10.1016/j.jclinepi.2023.03.022

[28] Keeley T, Williamson P, Callery P, Jones L, Mathers J, Jones J, et al. The use of qualitative methods to inform Delphi surveys in core outcome set development. Trials. 2016;17(1):230. 27142835 10.1186/s13063-016-1356-7 PMC4855446

[29] Üstün TB, Chatterji S, Bickenbach J, Kostanjsek N, Schneider M. The International Classification of Functioning, Disability and Health: a new tool for understanding disability and health. Disabil Rehabil. 2003;25(11–12):565–71. 12959329 10.1080/0963828031000137063

[30] Eroglu HS, Bowen A, Checketts M, Mitchell C. Managing Facial Palsy After Stroke: Results From an Online Survey of Health Professionals. Int J Lang Commun Disord. 2025;60(5):e70127. 40955872 10.1111/1460-6984.70127 PMC12439456

[31] Powell C. The Delphi technique: myths and realities. J Adv Nurs. 2003;41(4):376–82. 12581103 10.1046/j.1365-2648.2003.02537.x

[32] Lee A, Davies A, Young AE. Systematic review of international Delphi surveys for core outcome set development: representation of international patients. BMJ Open. 2020;10(11):e040223. 33234639 10.1136/bmjopen-2020-040223 PMC7684826

[33] Sinha IP, Smyth RL, Williamson PR. Using the Delphi technique to determine which outcomes to measure in clinical trials: recommendations for the future based on a systematic review of existing studies. PLoS Med. 2011;8(1):e1000393. 21283604 10.1371/journal.pmed.1000393 PMC3026691

[34] Barreto SR, Mourão AM, Chaves TS, Vicente LCC. The use of kinesio taping in the treatment of the acute phase of post-stroke facial paralysis. Audiol Commun Res. 2021;26:e2462.

[35] Kuttenreich AM, von Piekartz H, Heim S. Is There a Difference in Facial Emotion Recognition after Stroke with vs. without Central Facial Paresis? Diagnostics (Basel). 2022;12(7):1721. 35885625 10.3390/diagnostics12071721 PMC9325259

[36] Choi JB. Effect of neuromuscular electrical stimulation on facial muscle strength and oral function in stroke patients with facial palsy. J Phys Ther Sci. 2016;28(9):2541–3. 27799689 10.1589/jpts.28.2541 PMC5080171

[37] Guyatt GH, Oxman AD, Kunz R, Atkins D, Brozek J, Vist G, et al. GRADE guidelines: 2. Framing the question and deciding on important outcomes. J Clin Epidemiol. 2011;64(4):395–400. 21194891 10.1016/j.jclinepi.2010.09.012

[38] Brookes ST, Chalmers KA, Avery KNL, Coulman K, Blazeby JM. Impact of question order on prioritisation of outcomes in the development of a core outcome set: a randomised controlled trial. Trials. 2018;19(1):66. 29370827 10.1186/s13063-017-2405-6 PMC5784591

[39] Taylor E. We agree, don’t we? The Delphi method for health environments research. HERD. 2020;13(1):11–23. 31887097 10.1177/1937586719887709

[40] Kirkham JJ, Gorst S, Altman DG, Blazeby JM, Clarke M, Devane D, et al. Core outcome set–STAndards for reporting: the COS-STAR statement. PLoS Med. 2016;13(10):e1002148. 27755541 10.1371/journal.pmed.1002148 PMC5068732

[41] Wallace SJ, Worrall L, Rose T, Le Dorze G, Cruice M, Isaksen J, et al. Which outcomes are most important to people with aphasia and their families? An international nominal group technique study framed within the ICF. Disabil Rehabil. 2017;39(14):1364–79. 27345867 10.1080/09638288.2016.1194899

