# Supplementary material for: Towards the development of a core outcome set for post-stroke facial palsy (COS post-stroke facial palsy): a study protocol for establishing professional consensus on what to measure
Source: Trials. 2026 Mar 5;27:295. doi: 10.1186/s13063-026-09556-z (PMC13069702; doi:10.1186/s13063-026-09556-z)
Supplement: Supplementary file 2 — Supplementary file2. COS-STAP statement checklist (DOCX 20.0 KB) [file 13063_2026_9556_MOESM2_ESM.docx]

**Core Outcome Set-STandards Protocol Items: The COS-STAP Statement Checklist**

| **SECTION/TOPIC** | **ITEM No.** | **CHECKLIST ITEM** | **REPORTED ON PAGE NUMBER** |
| --- | --- | --- | --- |
| **TITLE/ABSTRACT** | | | |
| Title | 1a | Identify in the title that the paper describes the protocol for the planned development of a COS | Page 1, Line 1-3 |
| Abstract | 1b | Provide a structured abstract | Page 1 and 2, Line 20-60 |
| **INTRODUCTION** | | | |
| Background and objectives | 2a | Describe the background and explain the rationale for developing the COS, and identify the reasons why a COS is needed and the potential barriers to its implementation | Page 3, Line 67-110 |
|  | 2b | Describe the specific objectives with reference to developing a COS | Page 4 and 5, Line 116-135 |
| Scope | 3a | Describe the health condition(s) and population(s) that will be covered by the COS | Page 4, Line 129-132 and Page 4, Line 144-149 |
|  | 3b | Describe the intervention(s) that will be covered by the COS | Page 4, Line 144-149 |
|  | 3c | Describe the context of use for which the COS is to be applied | Page 4, Line 144-149 |
| **METHODS** | | | |
| Stakeholders | 4 | Describe the stakeholder groups to be involved in the COS development process, the nature of and rationale for their involvement and also how the individuals will be identified; this should cover involvement both as members of the research team and as participants in the study | Page 7, Line 214-217 |
| Information sources | 5a | Describe the information sources that will be used to identify the list of outcomes. Outline the methods or reference other protocols/papers | Page 6 and 7, Line 182-212 |
|  | 5b | Describe how outcomes may be dropped/combined, with reasons | Page 6 and 7, Line 182-212 |
| Consensus process | 6 | Describe the plans for how the consensus process will be undertaken | Page 9-11, Line 258-352 |
| Consensus definition | 7a | Describe the consensus definition | Page 9 Line 267-277 |
|  | 7b | Describe the procedure for determining how outcomes will be added/combined/dropped from consideration during the consensus process | Page 9-10, Line 277-292 |
| **ANALYSIS** | | | |
| Outcome scoring/feedback | 8 | Describe how outcomes will be scored and summarised, describe how participants will receive feedback during the consensus process | Page 9, Line 261-263  Page 10, Line 285-292  Page 11, Line 337-343 |
| Missing data | 9 | Describe how missing data will be handled during the consensus process | Page 9, Line 264-266 |
| **ETHICS and DISSEMINATION** | | | |
| Ethics approval/informed consent | 10 | Describe any plans for obtaining research ethics committee/institutional review board approval in relation to the consensus process and describe how informed consent will be obtained (if relevant) | Page 5-6, Line 154-163 |
| Dissemination | 11 | Describe any plans to communicate the results to study participants and COS users, inclusive of methods and timing of dissemination | Page 12, Line 2354-364 |
| **ADMINISTRATIVE INFORMATION** | | | |
| Funders | 12 | Describe sources of funding, role of funders | Page 14, Line 436-439 |
| Conflicts of interest | 13 | Describe any potential conflicts of interest within the study team and how they will be managed | Page 14, Line 430-432 |
